# Comparing Zinc Finger Nucleases and Transcription Activator-Like Effector Nucleases for Gene Targeting in Drosophila

**DOI:** 10.1534/g3.113.007260

**Published:** 2013-10-01

**Authors:** Kelly J. Beumer, Jonathan K. Trautman, Michelle Christian, Timothy J. Dahlem, Cathleen M. Lake, R. Scott Hawley, David J. Grunwald, Daniel F. Voytas, Dana Carroll

**Affiliations:** * Department of Biochemistry, University of Utah School of Medicine, Salt Lake City, Utah 84112; † Department of Genetics, Cell Biology and Development and Center for Genome Engineering, University of Minnesota, Minneapolis, Minnesota 55455; ‡ Mutation Generation and Detection Core, University of Utah Health Sciences Center, Salt Lake City, Utah 84112; § Stowers Institute for Medical Research, Kansas City, Missouri 64110; ** Department of Molecular and Integrative Physiology, University of Kansas Medical Center, Kansas City, Kansas 66160; †† Department of Human Genetics, University of Utah School of Medicine, Salt Lake City, Utah 84112

## Abstract

Zinc-finger nucleases have proven to be successful as reagents for targeted genome manipulation in *Drosophila melanogaster* and many other organisms. Their utility has been limited, however, by the significant failure rate of new designs, reflecting the complexity of DNA recognition by zinc fingers. Transcription activator-like effector (TALE) DNA-binding domains depend on a simple, one-module-to-one-base-pair recognition code, and they have been very productively incorporated into nucleases (TALENs) for genome engineering. In this report we describe the design of TALENs for a number of different genes in Drosophila, and we explore several parameters of TALEN design. The rate of success with TALENs was substantially greater than for zinc-finger nucleases , and the frequency of mutagenesis was comparable. Knockout mutations were isolated in several genes in which such alleles were not previously available. TALENs are an effective tool for targeted genome manipulation in Drosophila.

The advent of targetable DNA cleavage reagents has greatly enhanced the arsenal of tools for genetic and functional analysis. Zinc-finger nucleases (ZFNs) (Bibikova *et al.* 2002; Durai *et al.* 2005; Carroll 2011), homing endonucleases (Stoddard 2011), transcription activator-like effector nucleases (TALENs) (Christian *et al.* 2010; Cermak *et al.* 2011; Joung and Sander 2013), and CRISPR/Cas RNA−guided nucleases (Carroll 2012; Cho *et al.* 2013; Cong *et al.* 2013; Hwang *et al.* 2013; Jinek *et al.* 2013; Mali *et al.* 2013) can all be designed to cleave arbitrarily chosen, specific genomic DNA sequences. Repair of the induced breaks by cellular machinery leads to localized sequence changes via nonhomologous end joining (NHEJ), gene replacements via homologous recombination (HR) using an experimentally provided template, and other types of rearrangements (Porteus and Carroll 2005; Perez-Pinera *et al.* 2012; Piganeau *et al.* 2013).

The best of these reagents cleave their intended targets very efficiently, sometimes with frequencies exceeding 50% (Beumer *et al.* 2008; Cermak *et al.* 2011; Miller *et al.* 2011; Wood *et al.* 2011; Dahlem *et al.* 2012). This feature, plus the universality of DNA repair mechanisms, has made it possible to introduce novel mutations in essentially any gene in a wide variety of organisms (Carroll 2011; Joung and Sander 2013). ZFNs, in particular, have been used to modify the genomes of more than 20 different species (Gaj *et al.* 2013; Xiao *et al.* 2013) and they are currently being evaluated in human clinical trials (Urnov *et al.* 2010).

The DNA-binding module of ZFNs, the Cys_2_His_2_ zinc finger, contacts primarily three base pairs, and fingers have been identified that recognize most of the 64 different triplets (Dreier *et al.* 2000, 2001, 2005; Mandell and Barbas 2006; Thibodeau-Beganny *et al.* 2010; Sander *et al.* 2011; Hermann *et al.* 2012). Researchers have been frustrated, however, by apparent context effects on this recognition, *i.e.*, a particular finger will provide affinity and specificity for a given triplet in one sequence but not in others. Thus, combinatorial assembly of pre-existing fingers has met with only modest success (Sander *et al.* 2010; Thibodeau-Beganny *et al.* 2010). In addition, target-driven selection procedures for new finger combinations typically are laborious and uncertain of success (Thibodeau-Beganny and Joung 2007; Maeder *et al.* 2009).

TALENs use DNA-recognition modules that recognize single base pairs, linked to the same *Fok*I-derived cleavage domain that is used in ZFNs (Gaj *et al.* 2013; Joung and Sander 2013). Natural TALE proteins have several different modules for each of the four base pairs, but a code has been developed based on the most common modules, and this allows simple and effective assembly of new binding domains (Boch *et al.* 2009; Moscou and Bogdanove 2009). Continuing experience has led to a set of loosely defined standards for constructing TALENs, with generous ranges for the number of modules and the spacer between binding sites. Even the requirement for a T in the 5′ position of each binding site has been violated in at least one successful TALEN pair (Miller *et al.* 2011). Reports of successful applications to genomic targets are appearing at an accelerating rate (Bedell *et al.* 2012; Li and Yang 2013; Xiao *et al.* 2013).

In this study, we set out to test the efficacy of TALENs as gene targeting tools in Drosophila and to make direct comparisons with ZFNs that we previously characterized. Like others (Liu *et al.* 2012), we find that TALENs are frequently very effective, more reliably than modularly assembled ZFNs. Not every TALEN pair works, however, for reasons that are not evident from simple examination.

## Materials and Methods

### Stocks

Two different stocks were used for embryo injections. Canton-S was used as a wild-type for most injections. mRNAs with a donor were also injected into *w^1118^*, *P{EP}Lig4EP385*, a stock that carries a mutation in the *ligase 4* gene (Bloomington Stock Center, Bloomington, IN). New mutants in autosomal genes other than *ry* were collected by crossing to either *w^1118^* ; *Sco/S2CyO* or *TM2,rySC/MKRS* and collecting flies carrying a targeted chromosome over a balancer chromosome. A variety of stocks were used to characterize TALEN-generated mutations. All were obtained from the Bloomington Stock Center. The deletion stock *w^1118^*; *Df(2L)BSC295/CyO* was used to characterize mutations in *Psf2* (*CG18013*). Mutations in *Sld5* were characterized by crossing to *w^1118^*; *Df(3R)BSC495/TM6C*, *Sb^1^ cu^1^*. Mutations in *y* were scored by crossing to *C(1)DX*, *y^1^ f^1^/FM6*. Potential *ry* mutants were scored by crossing to *v^1^;ry^506^*. All stocks are described in FlyBase (Marygold *et al.* 2013). Before the final TALEN design, the candidate targets were amplified from the injection stock and sequenced. This is an important step, as differences from the reference sequence were often uncovered.

### TALEN plasmids

Several TALEN pairs were generated in the Voytas lab (Cermak *et al.* 2011). Two sets targeting *ry*—ryT1 and ryT2—and two targeting *y*—yT1 and yT2—were tested both in a full-length scaffold, with 231 amino acids in the linker between the TALE repeats and the nuclease domain, and a truncated version with a 63-amino acid linker. An additional two pairs, Sld5A and Sld5B were only used in the truncated scaffold. All others, targeting *ry*, *Psf2*, *Pcd*, *CG1220*, *CG7224*, *CG11594*, listed in Table 5, were constructed at the University of Utah Mutation Generation and Detection Core as described (Dahlem *et al.* 2012).

### Injections

RNAs were prepared and injected as described for ZFNs (Beumer *et al.* 2008), with the exception that TALEN plasmids generated in the Voytas lab were linearized with *Sac*I-High fidelity (New England Biolabs) and transcribed with MEGAscript T3 high yield transcription Kit (Ambion) and then capped with the ScriptCap m^7^G Capping System (CELLSCRIPT). RNAs were injected at concentrations from 0.2 to 0.4 mg/mL. Oligonucleotide donors generally were injected at 0.5 mg/mL, but in the case of the Psf2 phospho-oligo, injections were also done at 0.2 mg/mL. The *ry* donors used in this work were all single-stranded oligonucleotides obtained from the University of Utah DNA/peptide Core. The Psf2 donor was a single-stranded oligonucleotide obtained from Integrated DNA Technologies (Supporting Information, Table S1).

### Analysis of mutations

Mutations in the *ry* and *y* genes were analyzed by phenotype in the F1 generation and molecular analysis as previously described (Beumer *et al.* 2008). In summary, the sequence was amplified by polymerase chain reaction (PCR) with flanking primers, then sequenced at the University of Utah Sequencing Core. The primers used in each case are listed in Table S1. In HR experiments, the HR products were identified by amplifying appropriate fragments with fluorescently labeled primers, which were then run on an Applied Biosystems 3130xl capillary electrophoresis instrument (36 cm capillary, POP 7 polymer) and analyzed with Applied Biosystems GeneMapper software v 3.7. Donor-specific products were distinguished from wild-type based on a 1-bp deletion, then confirmed by sequencing, as described previously. Deletions generated between Try1 and Try3 were identified by PCR with ry-7100-F and ry-9532-R (Table S1). Wild type chromosomes generate a band of 3 kb, whereas deletions generate bands of ~490 bp. The latter were sequenced as described previously.

Mutations in *Pcd* (autosomal) and *CG12200* (X-linked) were screened by standard PCR and sequencing by selecting six F1 animals from each fertile G0 that had been previously crossed to an appropriate balancer. In brief, DNA was extracted from single flies (Gloor *et al.* 1993), and each target was amplified and sequenced (SIMR Molecular Biology Core Facility), using the forward primer for each (Table S1). *Pcd* and *CG12200* heterozygous females were identified as those whose sequence lost coherence at the target site, whereas CG12200 mutant males were identified by sequence. When a mutation was identified in any of the six F1 progeny, 30 individual lines were established from the brothers and/or sisters of the original fly by standard methods and then screened as before to identify the individual lines that carried the mutation.

Mutations in all other genes were scored as follows: Injected (G0) flies were collected and crossed to an appropriate stock as described previously. In 5−7 d, the G0 flies were recovered and DNA extracted as described (Beumer *et al.* 2006). Each G0 fly was then subjected to a high-resolution melting assay (HRMA; Figure S1) (Dahlem *et al.* 2012). All fertile vials from G0 flies that tested positive for mutant chromosomes by HRMA were collected and the remainder discarded. F1 progeny were then collected and crossed to an appropriate balancer stock. The flies were allowed to mate and lay eggs for an additional 5−7 d and again recovered and the DNA extracted. Another HRMA was performed, and all vials from progeny that tested as wild type were discarded (Figure S1B). F2 flies carrying balancers were collected from vials scored as heterozygotes and stocks established. Mutations were confirmed by sequencing at the University of Utah Sequencing Core, over a deletion if possible. All primers used in these assays are listed in Table S1.

## Results

To begin to understand how effective TALENs might be in Drosophila, we designed TALEN pairs to two genes previously targeted by well-characterized ZFNs (Bibikova *et al.* 2002, 2003; Beumer *et al.* 2006, 2008). In addition to making direct comparisons of efficacy, we wanted to address several aspects of TALEN function: (1) What TALEN scaffold works best in flies? (2) What nuclease architecture performs best in TALENs? (3) What is the nature of TALEN-induced mutations? (4) Will TALENs stimulate homologous recombination as well as ZFNs? (5) Can two TALEN pairs directed to separate locations in the same gene create large deletions? (6) How effectively can new TALENs be designed for new genomic targets?

### TALEN-ZFN comparisons and TALEN scaffolds

**Figure 1:**
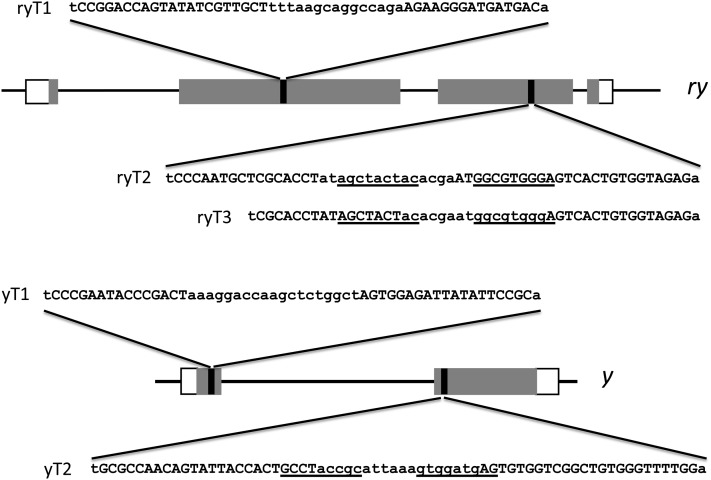
TALEN targets in the Drosophila *ry* and *y* genes. Each gene is diagrammed approximately to scale, with rectangles denoting exons and coding sequences as shaded rectangles. The locations of the TALEN targets are shown with black vertical lines, and the corresponding sequences are illustrated. TALEN binding sites are in capital letters, spacers in lower case. The ZFN binding sites that overlap the ryT2, ryT3, and yT2 sites are underlined.

TALENs were designed to two sites in each of two Drosophila genes, *rosy* (*ry*) and *yellow* (*y*). In each case, one site overlapped a sequence that had been successfully targeted with ZFNs (ryT2 and yT2; Figure 1). This allowed a direct comparison of activities. A second TALEN site was chosen arbitrarily in an exon of each gene (ryT1 and yT1). Coding sequences for each of the TALENs were produced, cloned, and transcribed *in vitro*. Appropriate pairs of these mRNAs were coinjected into Drosophila embryos with the use of standard procedures. Double-strand breaks made at the target sites often are repaired inaccurately by NHEJ, leaving small insertions, deletions, and substitutions at the cut site (Bibikova *et al.* 2002; Bozas *et al.* 2009). Mutations of this sort, particularly when they generate frameshifts, were expected to be nulls at all four sites. Therefore, germline mutations were identified by crossing the G0 (injected) flies, after they eclosed, to known *ry* or *y* mutants and scoring for the corresponding mutant phenotype—rosy eyes or yellow body color.

TALEN constructs are known to require some additional sequence from the natural TALE protein between the cluster of DNA-binding modules and the *Fok*I nuclease domain (Miller *et al.* 2011). Our initial constructs had long interdomain linkers of 231 amino acids. Each of the TALENs was subsequently modified to carry the 63-amino acid linker that had previously been shown to support improved cleavage activity (Miller *et al.* 2011; Dahlem *et al.* 2012). The results of assaying NHEJ mutagenesis with each of these eight TALEN pairs are shown in Table 1.

**Table 1:** NHEJ mutagenesis with TALENs and ZFNs

Nucleases	Linker (aa)	Parents	Yielders	Mutants	Mutants/Parent
ryT1	231	220	23 (10%)	93	0.42
	63	71	21 (30%)	685	9.65
ryT2	231	396	13 (3%)	43	0.11
	63	88	5 (6%)	88	1.00
yT1	231	194	14 (7%)	103	0.53
	63	81	39 (48%)	1329	16.41
yT2	231	93	0	0	0
	63	69	0	0	0
ryAB ZFNs	4	133	29 (22%)	632	4.75

TALENs for the ry and y genes are named as in Figure 1 and in the text. Two different lengths (in amino acids) of linker between the binding and cleavage domains were used for each TALEN pair. The Parents column shows the number of injected flies that were crossed to assess mutagenesis, and those that produced mutant offspring are shown as Yielders, with the percent of all parents they represent. The total number of mutants and the calculated number of mutants per parent are given. Results of an experiment done in parallel using the ryAB ZFNs are presented in the bottom line for comparison. NHEJ, nonhomologous end joining; TALEN, transcription activator-like effector nucleases; ZFNs, zinc-finger nucleases.

With the longer protein linker, TALEN pairs for three of the target sites induced germline mutations at modest frequencies (averages of 0.1–0.5 mutants per parent). Both the proportion of G0 flies that yielded mutants and the number of mutants were much lower than seen in a parallel experiment with ZFNs for the *ry* target. Nonetheless, assuming the average number of offspring to be approximately 80, the numbers of mutants represented between 0.14% and 0.66% of all F1 flies.

For each pair, truncation of the linker led to substantially greater mutation frequencies (Table 1). In the case of yT1, nearly half the G0 parents produced mutant offspring, and 20% of all F1s were mutant, an increase of more than 30-fold over the longer linker. An increase of more than 20-fold was seen for the ryT1 TALENs. The ryT2 pair, however, which targets a site that is cleaved efficiently by the ryAB ZFNs, was much less effective, even though the truncated linker enhanced its activity ninefold.

One TALEN pair, yT2, was ineffective at inducing new mutations with either linker (Table 1). We found it surprising that this particular pair failed, since its target overlaps a sequence that was effectively cut by ZFNs (Bibikova *et al.* 2002). The problem seems to be inherent in the TALEN design, as this pair also showed very low activity in a yeast assay (data not shown).

Interestingly, somatic mutations were observed directly in some G0 animals that were injected with the most active TALEN pairs. Approximately 45% of the yT1-63−injected G0 adults were predominately yellow throughout their cuticle, wings and bristles. These parents accounted for 69% of all the mutant progeny in the F1 generation; however, there were six such animals that produced no mutant progeny, so somatic phenotype is not a perfect indicator of germline mutagenesis. Surprisingly, the phenotypically *y* G0 animals were fairly evenly divided between males and females. As the *y* gene is on the X chromosome, it is easy to account for the *y* males, but the *y* females required biallelic disruption in somatic cells. The yT1 TALEN pair is clearly very active.

It is even more surprising that we observed G0 animals with rosy eyes in the ryT1-63 injections. The *ry* gene is not expressed in the eye. Its product, xanthine dehydrogenase, is synthesized in other tissues and transported into the eye (Reaume *et al.* 1989). Furthermore, the *ry* gene needs to be expressed at only about 10% of the wild-type level to produce the wild-type phenotype (Keller and Glassman 1964). Because it is autosomal, this phenotype also requires biallelic disruption. Nonetheless, we observed 5–10% of the G0 flies displaying a mutant phenotype.

In summary, three of four different TALEN designs generated useful frequencies of NHEJ mutations in the Drosophila *ry* and *y* genes. The lowest frequencies were obtained with pairs directed to sequences previously targeted by ZFNs, with better frequencies. TALENs for the other site in each gene were very active. As seen by others TALENs with a 63-aa linker between the binding and cleavage domains were substantially more active than those with a much longer linker (Miller *et al.* 2011; Dahlem *et al.* 2012; Joung and Sander 2013).

### Wild-type *vs.* obligate heterodimer nucleases

We next addressed the issue of the architecture of the nuclease domain in both ZFNs and TALENs. Several groups have introduced sequence changes in the *Fok*I dimer interface that prevent homodimerization while allowing the necessary heterodimerization (Miller *et al.* 2007; Szczepek *et al.* 2007; Doyon *et al.* 2011; Ramalingam *et al.* 2011). This maneuver sharply reduces toxicity that can often be attributed to homodimerization of one of the nuclease pair at sites related, but not identical, to its supposed target (Bibikova *et al.* 2002; Beumer *et al.* 2006). Variable results have been reported regarding consequences of these changes on cleavage activity, with some investigators finding a significant drop in activity, whereas others report minimal effects (Miller *et al.* 2007; Doyon *et al.* 2011).

We first tested obligate heterodimer modifications described by Miller *et al.* (2007) for the ability to reduce the toxicity of two individual ZFNs. The yA nuclease is one of a pair targeting the *yellow* gene, whereas bwB is one of a pair for the *brown* gene. Their expression was induced with a 37° heat shock in larvae carrying the corresponding genes under control of an hsp70 promoter. Both the single substitution E490K and the double replacement E490K/I538K (KK) completely eliminated the lethality seen with each ZFN alone. With a single substitution in yA and the wild type cleavage domain in its partner, yB, rather good mutant yields were obtained (Table 2). With the KK double substitution in yA and with the complementary obligate heterodimer modifications in yB (KK/EL in Table 2), the mutant yield dropped significantly.

**Table 2:** Effects of obligate heterodimer modifications

Nucleases	RNA Conc.	Cleavage Domains	Parents	Yielders	Mutants	Mutants/ Parent
yAB ZFNs		wt/wt	(few survivors)			
		K/wt	63	24	241	3.82
		K/EL	144	31	31	0.22
		KK/EL	152	1	1	0.01
ryAB ZFNs	350	wt/wt	139	74	2126	15.3
	350	KK/EL	129	18	90	0.70
	600	KK/EL	143	23	67	0.47
	1000	KK/EL	64	17	85	1.33
	350, polyA	KK/EL	28	4	90	3.2
ryT3 TALENs	350	wt/wt	214	60	914	4.27
	350	DD/RR	100	4	14	0.14
	350	DDD/RRR	136	49	582	4.28

The experiments with the yAB ZFNs were performed by heat-shock induction in larvae. All other experiments were done by embryo injection. The indicated RNA concentration in the injection solution is in μg/mL. The last entry for the ryAB ZFNs had an extended poly A tail on both mRNAs. The cleavage domain modifications are: K, E490K; KK, E490K, I538K; EL, Q486E, I499L; DD, R487D; RR, D483R; DDD, R487D, N496D; RRR, D483R, H537R.

This effect was confirmed in embryo injection experiments with the ryAB ZFNs. This pair shows no evidence of toxicity, but its efficacy dropped sharply when the obligate heterodimer substitutions were introduced (Table 2). The loss of activity was not recovered by increasing the concentration of the injected mRNAs and only minimally regained by increasing the length of their polyA tails (Table 2).

Although none of the TALENs we have worked with showed overt toxicity as the result of nonspecific cutting as we saw with some ZFNs, the possibility still exists. Thus, we explored the same issues with a new pair of TALENs designed to the site in the *ry* gene targeted by the ryAB ZFNs. This pair, designated ryT3, differs slightly from the ryT2 pair (Figure 1). They contain the 63-amino acid linker and were constructed with three different cleavage domain architectures: wild type, single substitutions in each partner, R487D/D483R (DD/RR), and the double substitutions R487D, N496D/D483R, H537R (DDD/RRR) (Meng *et al.* 2008; Dahlem *et al.* 2012). As shown in Table 2, the pairs with the wild-type cleavage domains and the DDD/RRR pair gave very good yields of mutants, whereas the singly modified pair, DD/RR, was dramatically less effective. Both the wild-type and DDD/RRR ryT3 TALENs gave greater yields of mutants than the ryT2 pair, which carries wild-type cleavage domains, directed to essentially the same target. We do not know what feature of the nucleases or the target might account for this difference.

In summary, in Drosophila, the first generation obligate heterodimer modifications of the *Fok*I cleavage domain (KK/EL and DD/RR) are very effective in eliminating the toxicity of individual ZFNs. This comes with a substantial decrease in the efficiency of cleavage and mutagenesis, however. Efficacy is restored with the second generation modifications, DDD/RRR, at least in the context of TALENs, and likely for ZFNs as well.

### Nature of TALEN-induced mutations

Mutations arising from cleavage by both TALENs and ZFNs are caused by inaccurate repair by NHEJ. Despite relying on the same process, the two sources yield somewhat different spectra of sequence changes (Chen *et al.* 2013; Kim *et al.* 2013). Both show small insertions and deletions at the break site. In the case of TALENs, two-thirds of all mutations in our sample were simple deletions, nearly all the rest were deletions accompanied by insertions, while only a single example of a simple insertion was recovered (Table 3). In contrast, about half the ZFN-induced mutations were simple deletions, and the remainder was distributed equally between simple insertions and insertions with deletions.

**Table 3:** Comparison of TALEN- and ZFN-induced mutations

Mutation	ZFNs	TALENs
Simple deletion, %	51	68
Deletion w/insertion, %	24	30
Simple insertion, %	24	1
Total number	632	148

Data are tabulated for all the successful TALEN pairs listed in Table 3 and for the ZFN pairs ryAB and yAB, and are rounded to the nearest whole percent.

**Figure 2:**
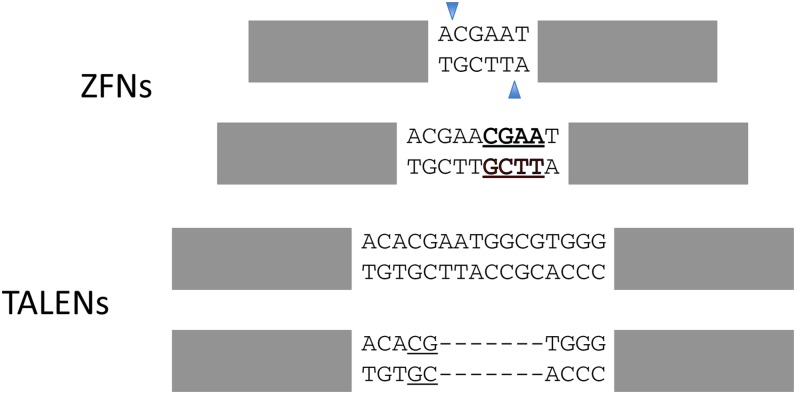
The most common single mutations found at overlapping ZFN and TALEN targets in *ry* exon 3 (ryAB ZFNs, ryT3 TALENs). Gray rectangles denote the binding sites for the DNA-binding modules; the spacer sequences are written out. The most common ZFN product is an apparent fill-in and blunt join of the 4-nt 5′ overhang created by cleavage: the duplicated 4 bp are underlined and in bold. The most common TALEN product is a 7-bp deletion supported by a 2-bp microhomology (underlined).

A very common mutation recovered after ryAB ZFN expression was a 4-bp insertion that we attribute to fill-in and blunt joining of the 4-base 5′ overhang generated by cleavage (Figure 2). Insertions of this type were never seen among TALEN-induced mutations at any target, including the ones that overlap the ryAB site. In fact, the single most common mutation at the ryT3 site was a 7-bp deletion that may be mediated by a 2-bp microhomology (Figure 2).

**Figure 3:**
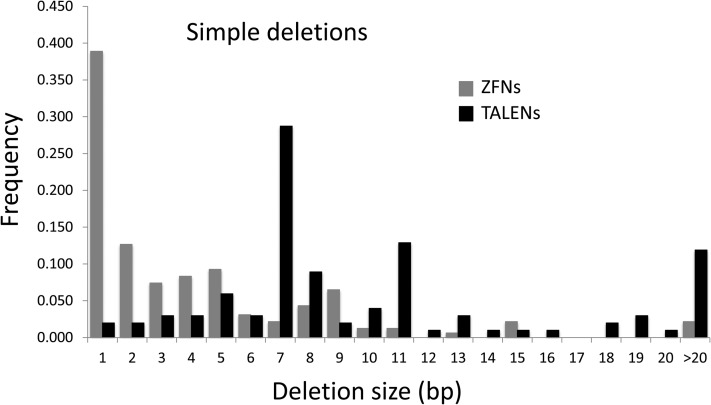
Distribution of deletion sizes for ZFNs and TALENs. The median deletion size was 2 bp for ZFNs and 8 bp for TALENs. The data for ZFNs reflect results for the *y* and *ry* targets (Bibikova *et al.* 2002; Beumer *et al.* 2006, 2008). The TALEN data include all sites presented in this study, with more examples from the *y* and *ry* targets than from others.

The distribution of deletion sizes also differed between ZFNs and TALENs. While both distributions were broad, the median was significantly larger in the case of TALEN-induced deletions (Figure 3). This was particularly true for simple deletions and less dramatically for ones accompanied by insertions (Figure S2). Insertion sizes differed only slightly between the nucleases (Figure S3).

### Inducing large deletions with TALENs

Having effective TALENs for two sites, separated by 2.5 kb, in the *ry* gene allowed us to test whether simultaneous expression of both pairs would induce deletions between them, as has been observed in cell lines with ZFNs (Lee *et al.* 2010b). Injections were performed with all four mRNAs in the same injection mix. Interestingly, the effects of the two TALEN sets appear to be synergistic, resulting in an average of 27 mutant progeny per scored parent, far more than the nine seen for ryT1 or the four seen for ryT3 (Table 4). Coinjection of the four individual mRNAs did not cause any apparent lethality, so coexpressing multiple TALEN pairs could be an effective method for simultaneously generating mutants in several targets.

**Table 4:** NHEJ mutagenesis with two TALEN pairs

Nucleases	Linker (aa)	Parents	Yielders	Mutants	Mutants/ Parent
ryT1 + ryT3	63	141	76 (54%)	3769	26.7

mRNAs for TALEN pairs ryT1 and ryT3 were mixed and coinjected into embryos. Entries are as in Table 1. NHEJ, nonhomologous end joining.

**Figure 4:**
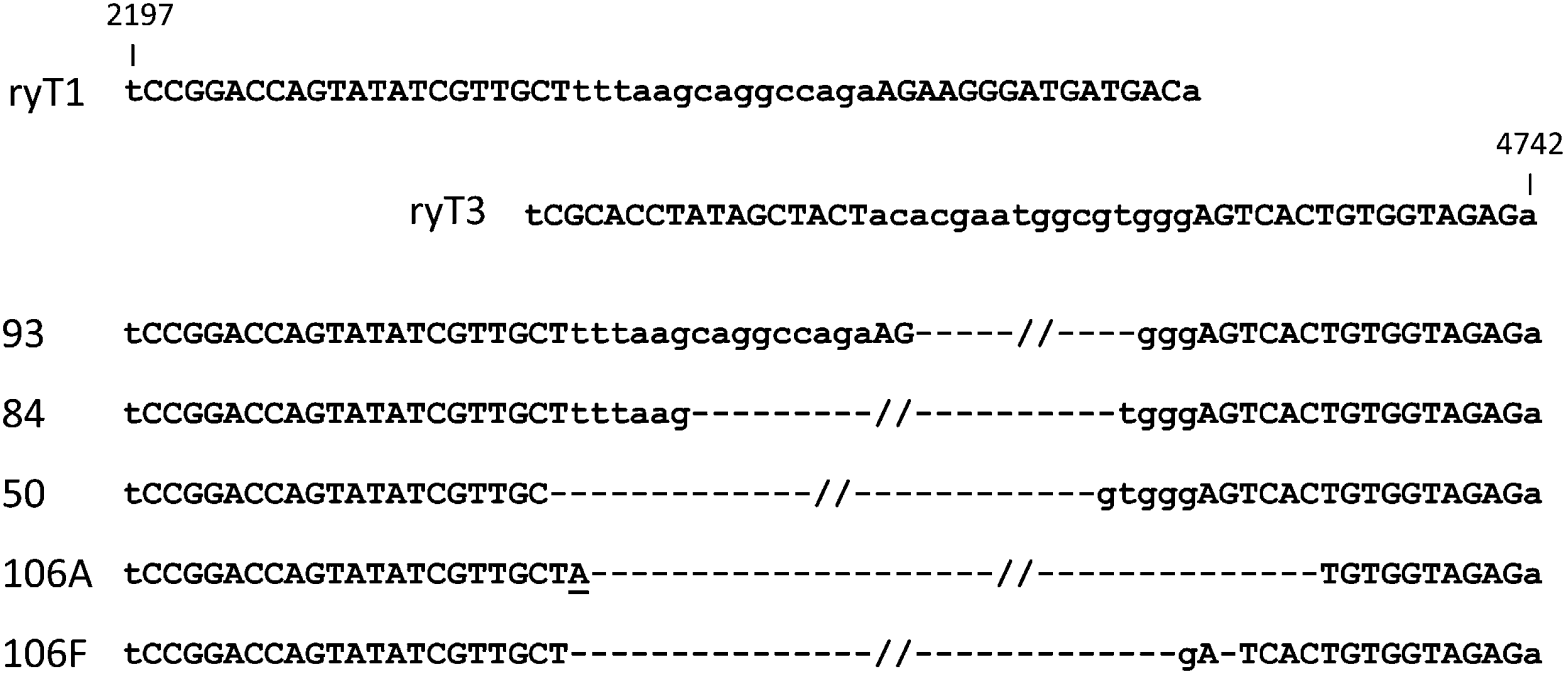
Sequences of the junctions of the 2.5-kb deletions created by coinjection of mRNAs for the ryT1 and ryT3 TALENs. The target sequences for the individual TALEN pairs are shown at the top; the positions of these sequences relative to the start of *ry* transcription are given. The sequences of the deletions, which have been given arbitrary numerical designations, are aligned to them below. As in Figure 1, TALEN binding sites are shown in capital letters, spacers in lower case. An apparent single-base substitution in deletion 106A is underlined.

To identify deletions in these mutants, PCRs were performed with primers flanking the expected deletion. After testing 384 mutant F1 animals, 12 (3%) mutations were scored as deletions. Six of these were sequenced, including two pairs of siblings. Five of the six deletions were unique, and all ten endpoints were within or just outside the TALEN spacers (Figure 4). Although only 3% of all *ry* flies, if all 3769 mutants had been screened, over 100 large deletions could have been recovered in this single experiment.

### Homologous recombination

**Figure 5:**
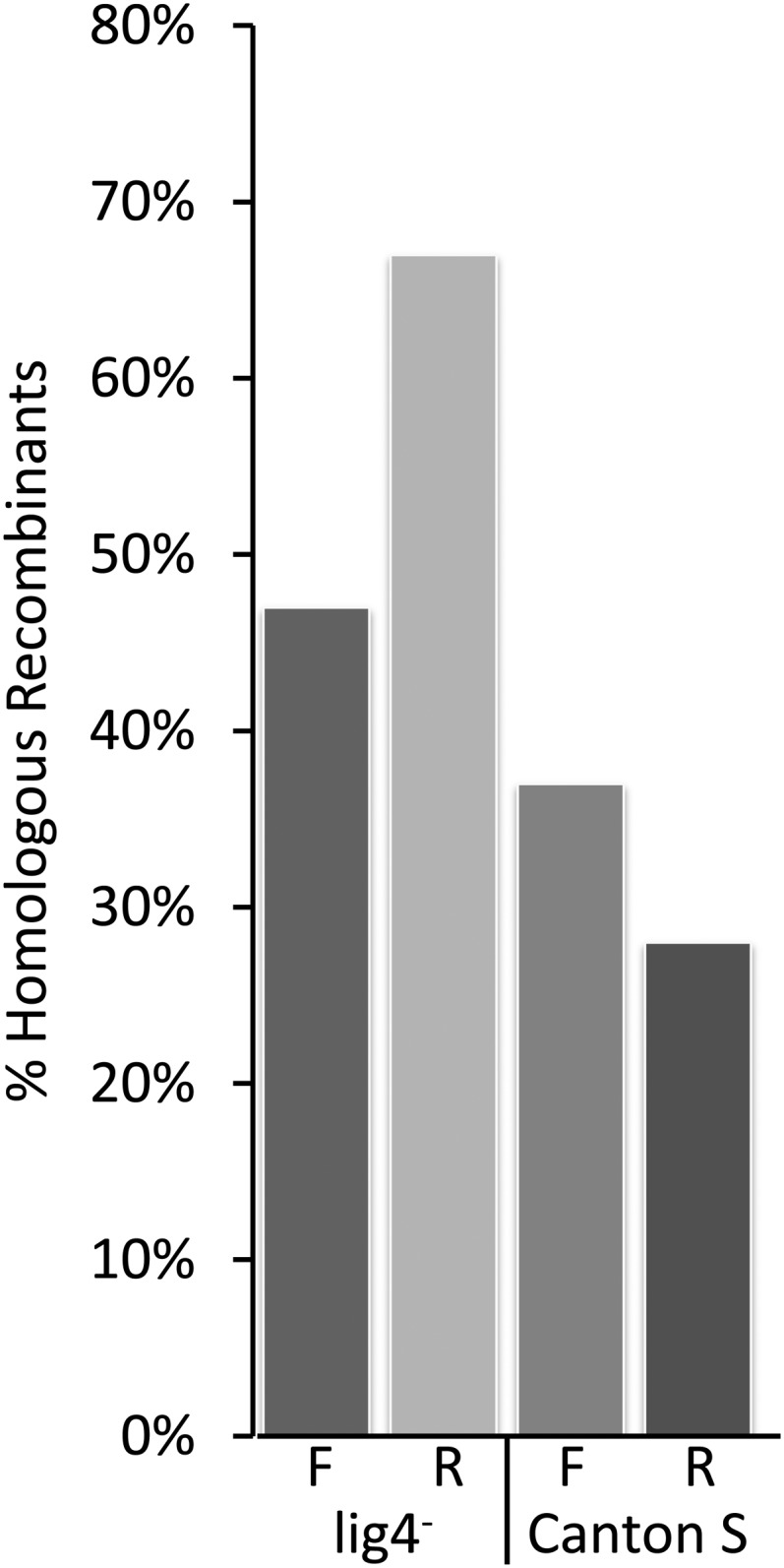
Histograms of the percent of mutant progeny scoring positive for homologous recombination achieved with oligonucleotide donors in conjunction with the ryT3 TALENs. Data are from experiments in a wild type strain (Canton S) and in a strain that lacks DNA ligase IV (lig4^−^).

Experiments to test the efficiency of TALEN-induced DSBs in stimulating HR at the ryT3 target were conducted in both wild-type and *lig4* mutant flies. The donors were 111-base single-stranded oligonucleotides originally constructed for use with ry ZFNs (Beumer *et al.* 2013) (Figure S4). These oligos carry a single G deletion creating a frameshift that inactivates the gene. The oligos also carry substitutions on either side of the cut site that are useful for determining how much donor sequence was incorporated into the target chromosome. We tested two oligos, one homologous to the forward strand, and the other the reverse complement (Figure S4). Both oligos served effectively as donors, with outcomes very similar to those seen for ZFN cleavage, including HR being greater in the *lig4* background than in wild-type flies (Figure 5) (Beumer *et al.* 2013). Conversion tracts were indistinguishable from those generated in the ZFN experiments and showed no bias based on the polarity of the oligo donor.

### Targeting additional genes

Our success with the easily scored *ry* and *y* genes encouraged us to design TALEN pairs for additional genes in which useful mutations had not previously been isolated. The target genes and properties of the TALENs are listed in Table 5, along with the ry and y TALENs for comparison. All these constructs contained the 63-aa linker and carried either the wild-type cleavage domain or the DDD/RRR obligate heterodimer modifications. The specific target sequences were chosen following the rough guidelines published previously (Cermak *et al.* 2011) and are listed in Table S2.

**Table 5:** TALEN parameters and activities

Gene	TALENs	L, bp	Spacer, bp	R, bp	Activity
*ry*	ryT1	21	15	15	+
	ryT2	16	15	26	+
	ryT3	16	16	16	+
*y*	yT1	15	19	19	+
	yT2	24	18	24	−
*Psf2*	A	21	14	18	+
	B	16	22	16	−
	C	15	17	16	+
	D	19	16	17	+
*Sld5*	A	16	15	27	+
	B	15	15	16	+
*Pcd*	A	18	18	20	+
	B	16	16	17	+
*CG12200*	A	21	17	20	+
	B	18	15	20	+
*CG7224*	A	18	18	17	+
*CG11594*	A	20	17	18	+

Multiple TALEN pairs were produced for the first 6 genes in the list. The numbers of base pairs in the left (L) and right (R) halves of each target are given, along with the length of the spacer between binding sites. Activity reflects whether (+) or not (−) mutants were obtained following injection of the indicated pair. The Psf2 A and B pairs have exactly the same R binding site and module composition, but differ in the L binding site and spacer. Numerical details are provided in Table 1 for *ry* and *y* TALENs, and in Table S3 for the others.

Mutations were readily obtained with all but two of the 17 TALEN pairs tested, including the ry and y TALENs (Table 5). It is difficult to make comparisons of the relative activities because the methods used to detect mutations differed substantially among target genes. With the exception of the *ry* and *y* targets, mutations at the other sites were identified by molecular analysis rather than phenotypic screening. Typically an initial screen was done by HRMA (see *Materials and Methods*) on a number of G0 flies to identify ones with somatic mutations and therefore more likely to produce mutant offspring (Dahlem *et al.* 2012) (Figure S1A). Offspring of these flies were analyzed individually by HRMA and by sequence analysis of PCR products that included the target site (Figure S1B). Details of these analyses are provided in Table S3.

TALENs for *Psf2* and *Sld5* were able to generate mutations effectively, as revealed by HRMA, but many of the G0 individuals died as pupae. We attribute this to the production of biallelic mutations in enough somatic cells to cause lethality. Reducing the concentration of TALEN mRNAs injected allowed us to recover viable adults, although many of these were sterile. It is also possible that off-target cleavage is responsible for these effects, although this was typically revealed as much earlier lethality in embryos or larvae with toxic ZFNs. This again highlights the effectiveness of TALENs.

For comparison, we present results of our attempts to target 10 Drosophila genes with ZFNs (Table S4). Five of the 10 genes were successfully mutated, as previously reported (Beumer *et al.* 2006, 2008), but nine of the 14 ZFN pairs failed. This was true, even though the specific targets had been carefully chosen to be rich in GNN triplets (Table S5), particularly ones with very promising *in vitro* binding properties (Carroll *et al.* 2006). Notably, two ZFN pairs for the *Sld5* gene and one for *CG7224* failed, whereas both genes were readily mutated with TALENs.

Among the two failed TALEN pairs, the one called Psf2B, which showed a very low level of mosaicism in the HRMA, almost certainly had a spacer that was too long (22 bp). The Psf2A pair that succeeded used exactly the same Right member of the pair but had a shorter spacer and a Left member that was not obviously a better design. The failure of the yT2 pair was quite surprising, because ZFNs for essentially the same target worked quite effectively (Bibikova *et al.* 2002). This finding suggests that some aspect of TALEN design was at fault, rather than a characteristic of the target sequence. Success in each case was determined simply by whether or not mutations were recovered, and it was not possible to set a standard limit of detection for all targets. Thus, it is quite conceivable that mutations could be recovered at the “failed” sites with more extensive screening.

We also attempted to recover mutants generated by HR at the Psf2A locus. The donor oligo, designated PSF2-trunc, was designed to create a truncation in the *Psf2* gene, by introducing a stop codon and a diagnostic restriction enzyme site. Two of the fertile G0 adults gave recombinant progeny, as determined by HRMA and restriction digest, and confirmed by sequencing. Thus, HR can effectively be used at loci other than the *ry* gene.

## Discussion

Like other researchers working in a variety of organisms, we find that TALENs are easy to design for new targets, the designs are quite often successful, and the frequency of induced mutation is remarkably high (Christian *et al.* 2010; Clark *et al.* 2011; Huang *et al.* 2011; Miller *et al.* 2011; Wood *et al.* 2011). In these respects, TALENs typically out-perform ZFNs, although not all TALENs work, and some ZFNs are equally or more effective, *e.g.*, the ryAB pair (Beumer *et al.* 2008; Chen *et al.* 2013).

When TALENs do not work at a useful level, it is difficult to know why. Failure could reflect a problem with TALEN design, with accessibility of the target sequence, or a delivery issue. Among the two pairs that failed in our experiments, one very likely was poorly designed, with a spacer between the TALE binding sites that was too long (Psf2B). Because other TALENs for this gene produced useful mutants, the failed pair was not pursued. It does not appear that any of the failures were due to the toxicity we have seen due to off-target cleavage with ZFNs. Although in some cases we saw lethality late in development, this was most likely due to biallelic disruption of an essential target gene. We saw no reduction in viability that could not be tied to the phenotype of the gene being targeted. This is consistent with results seen by others working with TALENs (Mussolino *et al.* 2011; Tesson *et al.* 2011; Cade *et al.* 2012; Qiu *et al.* 2013)

The most surprising failure was the yT2 TALEN pair. It was directed to the same site in the *yellow* gene that was successfully targeted with ZFNs (Bibikova *et al.* 2002). This strongly suggests that target accessibility is not the problem. Both of the yT2 TALEN monomers had unusually long DNA-binding domains, 24 modules on each side. It is possible that this creates problems for dimerization of the cleavage domain. Alternatively, the DNA ends may be bound by the proteins so avidly following cleavage, that processing and mutagenesis are inhibited. TALENs with long module arrays on one side readily yielded mutants, however (Table 5). The ryT2 pair produced mutations that deleted a short distance into the binding sites on both sides, suggesting that the 26-module TALEN did not block degradation or joining. The few mutations we characterized for the Sld5A pair were all confined to the spacer.

A feature of TALEN-induced mutations that has been described in other cell types as well is that they are biased toward deletions, in preference to insertions (Chen *et al.* 2013; Kim *et al.* 2013). The deletions, although still short (median = 8 bp), are significantly longer than those produced by ZFNs (median = 2 bp). We presume this reflects the longer spacers in the TALEN targets. It could be that degradation at nuclease-induced ends proceeds readily until the protein-bound sequences are approached, then slows; however, we see no evidence of preferred end points for deletions in most cases. Another explanation may be that larger deletions or insertions are required to render the target immune to recutting by the TALENs. A single-base insertion or deletion in the spacer quickly discourages additional ZFN cutting (Bibikova *et al.* 2001), but TALENs tolerate a much larger range of spacer sizes (Christian *et al.* 2010; Miller *et al.* 2011; Mussolino *et al.* 2011). It might also be that TALENs sometimes cut more than once in the spacer at their targets. Although the first DSB should allow the ends to separate, it is possible that the TALENs do not release the ends immediately.

We were also surprised not to find products that correspond to a simple 4-base fill-in and blunt join among any of the TALEN mutations we sequenced. This was quite a common product of ryAB ZFN cleavage (Beumer *et al.* 2006; Beumer *et al.* 2008). We presume that the *Fok*I cleavage domain produces the same end configuration whether it is linked to zinc fingers or to TALE modules. The difference could again reflect the longer, more flexible spacer requirements for TALENs, either more rapid degradation or multiple cuts, as discussed above.

The generation of chromosomal deletions and, potentially, other rearrangements, is of particular interest to the fly community. Similar techniques have been used in cell lines to generate deletions, duplications, inversions and translocations (Lee *et al.* 2010a,b, 2012; Piganeau *et al.* 2013). Although some of these studies have used ZFNs rather than TALENs, it appears that the efficiency of the nucleases involved is the critical factor. Thus, given the ease of TALEN design allowing precise placement of rearrangement endpoints, we expect that this use of TALENs will be particularly productive.

In conclusion, we have found TALENs to be very effective agents for germline mutagenesis in Drosophila. We were able to produce targeted mutations in eight different genes, six of which had no previously described alleles. The mutation frequency was high enough that straightforward molecular analysis of G0 and F1 flies was adequate to identify and isolate the desired mutants. The TALEN platform has clear advantages over ZFNs, including ease of construction and a higher success rate. The emerging CRISPR/Cas RNA-guided nuclease technology has its own favorable characteristics, including ease of generating reagents, simple multiplexing, and high efficiency; and it has been applied successfully to Drosophila (Bassett *et al.* 2013; Gratz *et al.* 2013; Yu *et al.* 2013). However, there is some indication that the RNA-guided nucleases are inherently less specific than TALENs (Cong *et al.* 2013; Fu *et al.* 2013). It will be interesting to see how each of these approaches develops. It is safe to say that the tools for reverse genetics have become very powerful, both in their ease of use and in their application to a wide variety of cells and organisms.

## Supplementary Material

Supporting Information
