# Supplementary material for: Comparing Zinc Finger Nucleases and Transcription Activator-Like Effector Nucleases for Gene Targeting in Drosophila
Source: G3 (Bethesda). 2013 Oct 1;3(10):1717–25. doi: 10.1534/g3.113.007260 (PMC3789796; doi:10.1534/g3.113.007260)
Supplement: Supporting Information [file supp_3_10_1717__index.html]

Comparing Zinc Finger Nucleases and Transcription Activator-Like Effector Nucleases for Gene Targeting in Drosophila — Supporting Information 

# Comparing Zinc Finger Nucleases and Transcription Activator-Like Effector Nucleases for Gene Targeting in Drosophila

## Supporting Information for Beumer *et al.*, 2013

**Files in this Data Supplement:**

- Supporting Information - Figures S1-S4 and Tables S1-S5 (PDF, 438 KB)
- Figure S1 - Detecting mutants in *Psf2* with HRMA after mutagenesis with the TALEN pair Psf2A (PDF, 96 KB)
- Figure S2 - Distribution of the sizes of deletions that are associated with insertions, for ZFNs and TALENs (PDF, 131 KB)
- Figure S3 - Distribution of the sizes of insertions for ZFNs and TALENs (PDF, 148 KB)
- Figure S4 - Sequences of oligonucleotides used as donors with ryT3 TALENs (PDF, 67 KB)
- Table S1 - Oligonucleotides used in this paper (PDF, 53 KB)
- Table S2 - TALEN target sequences (PDF, 42 KB)
- Table S3 - Details of TALEN mutagenesis (PDF, 57 KB)
- Table S4 - ZFN parameters and activities (PDF, 56 KB)
- Table S5 - ZFN target sequences (PDF, 63 KB)
