# Supplementary material for: Comparing Zinc Finger Nucleases and Transcription Activator-Like Effector Nucleases for Gene Targeting in Drosophila
Source: G3 (Bethesda). 2013 Oct 1;3(10):1717–25. doi: 10.1534/g3.113.007260 (PMC3789796; doi:10.1534/g3.113.007260)
Supplement: Supporting Information [file supp_g3.113.007260_007260SI.pdf]

G3: Genes | Genomes | Genetics

Comparing ZFNs and TALENs for Gene Targeting in Drosophila

Kelly J. Beumer[1], Jonathan K. Trautman[1], Michelle Christian[2], Timothy J. Dahlem[3], Cathleen M. Lake[4], R. Scott Hawley[4,5], David J. Grunwald[6], Daniel F. Voytas[2] and Dana Carroll[1,*]

[1]Department of Biochemistry, University of Utah School of Medicine, Salt Lake City, UT

[2]Department of Genetics, Cell Biology and Development and Center for Genome Engineering, University of Minnesota, Minneapolis, MN

[3]Mutation Generation and Detection Core, University of Utah Health Sciences Center, Salt Lake City, UT

[4]Stowers Institute for Medical Research, Kansas City, MO

[5]Department of Molecular and Integrative Physiology, University of Kansas Medical Center, Kansas City, KS

[6]Department of Human Genetics, University of Utah School of Medicine, Salt Lake City, UT

*Corresponding author

DOI: 10.1534/g3.113.007260

A

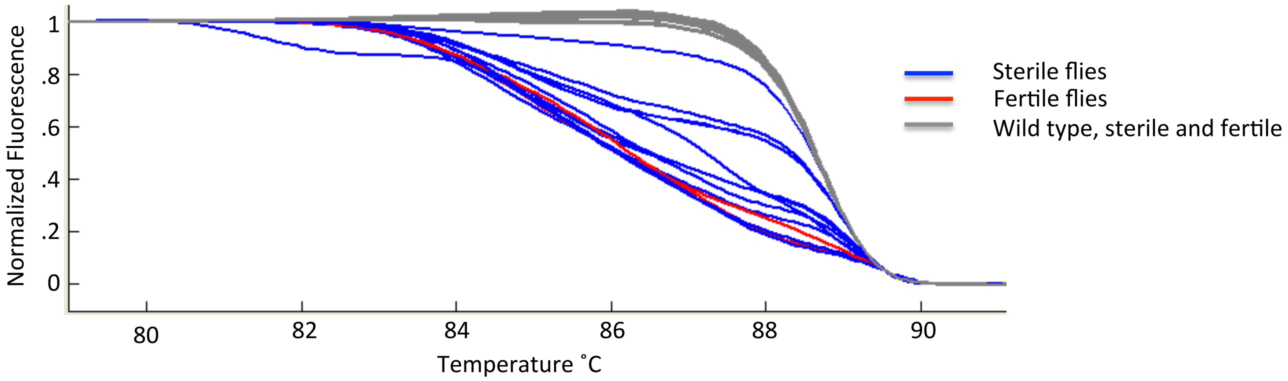

B

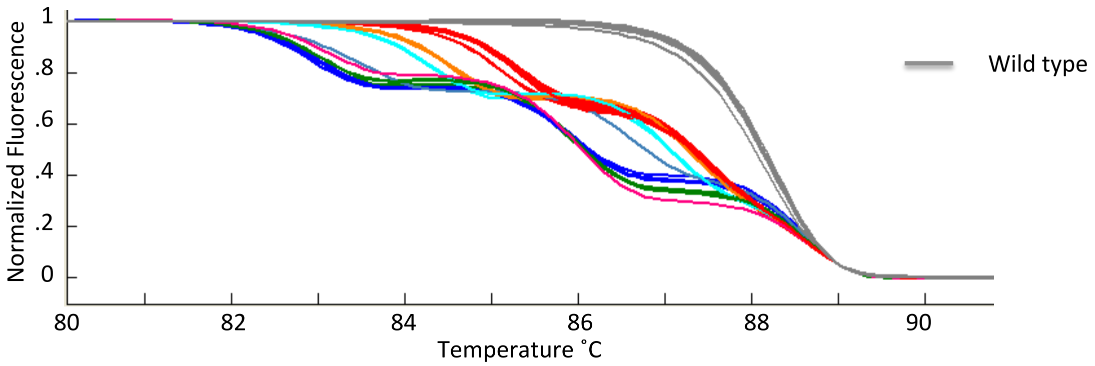

Figure S1 Detecting mutants in *Psf2* with HRMA after mutagenesis with the TALEN pair Psf2A. A. G0 flies were crossed to a balancer stock, allowed to lay for 5-7 days, then collected and tested, along with 8 known wild types. All flies that do not group with the known wild types are considered mutants. In this case, many were sterile, so the 2 fertile vials were kept, and the remainder discarded. All males and virgins from these vials were collected and crossed. B. After being allowed to lay for 5-7 days, the F1 flies were collected and tested in the same way. Each different color represents a unique mutant heterozygote.

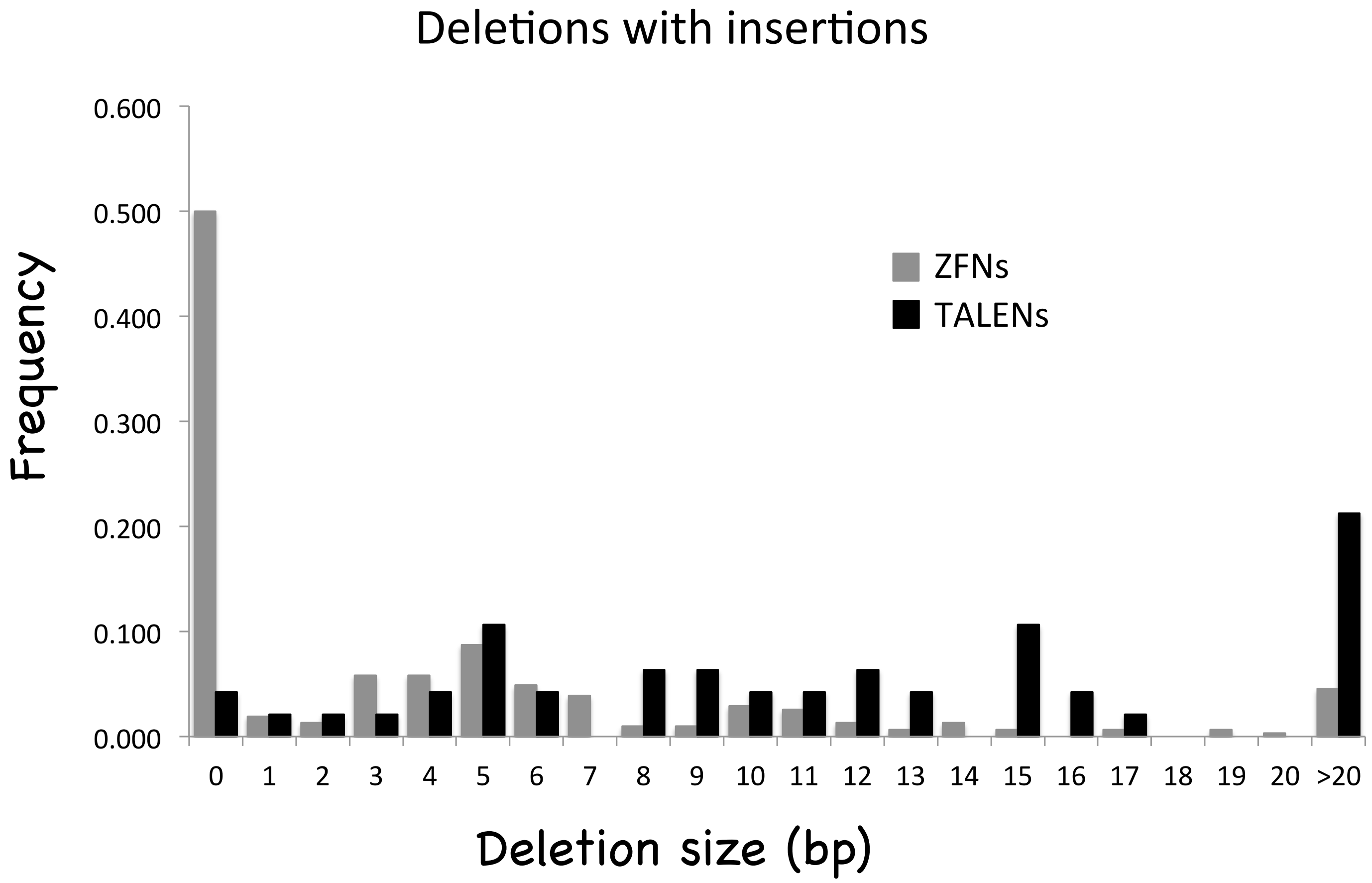

Figure S2 Distribution of the sizes of deletions that are associated with insertions, for ZFNs and TALENs. Data as in Figure 3 of the main text.

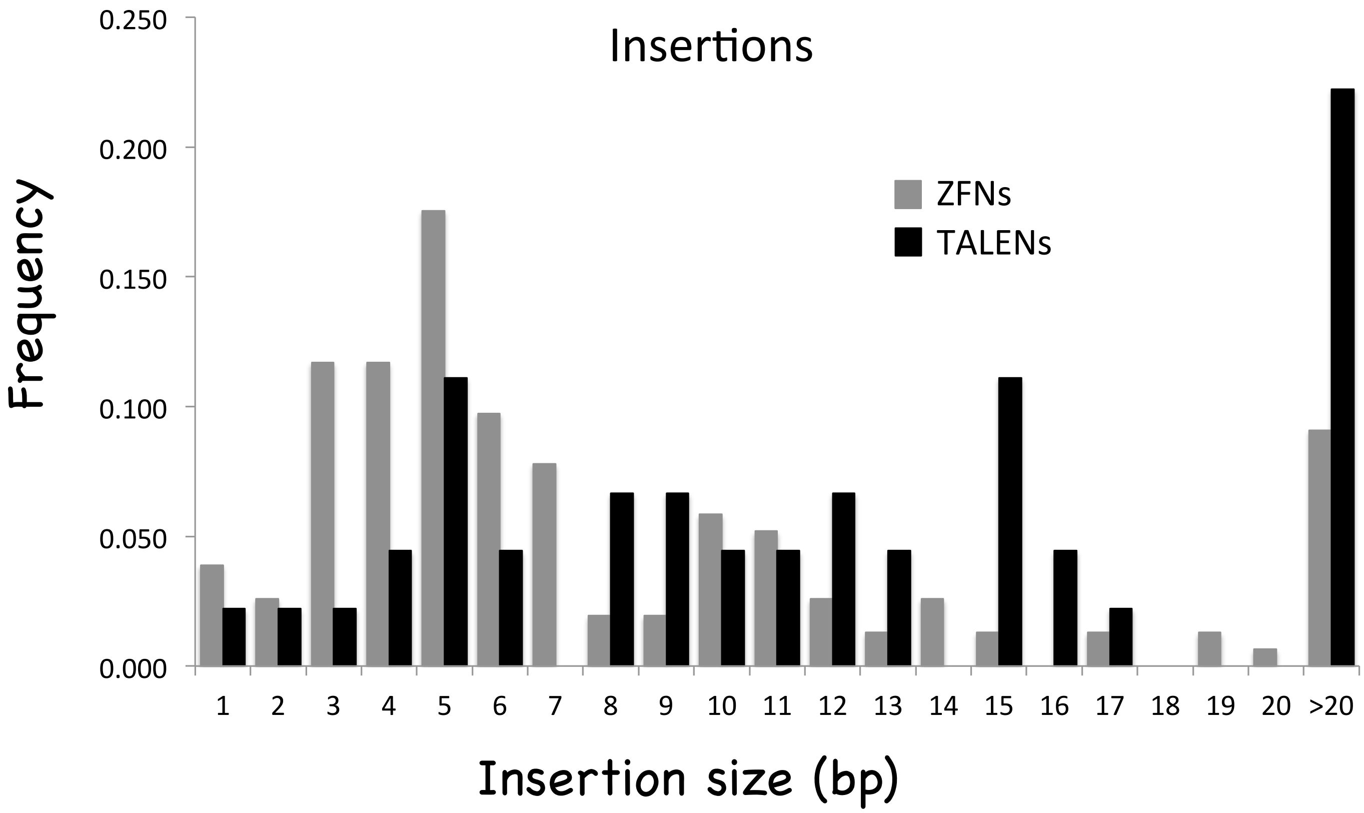

Figure S3 Distribution of the sizes of insertions for ZFNs and TALENs. Data as in Figure 3 of the main text.

Oligo F *Eco*RI

5'-TTGGATATCACCCGGAAACGAATTCCAATGCC**CGCACCTATAGCTACT**ACAC_AATGGCGT
GGG**AGTCACTGTGGTAGCG**ATCGATTGCCTGACTGGCGACCATCAGGTGCT-3'

*Pvu*I

Oligo R

5'-AGCACCTGATGGTCGCCAGTCAGGCAATCGAT**CGCTACCACAGTGACT**CCCACGCCATT_G
TGT**AGTAGCTATAGGTGCG**GGCATTGGAATTCGTTTCCGGGTGATATCCAA-3'

Figure S4 Sequences of oligonucleotides used as donors with ryT3 TALENs. Each sequence starts at the 5' end. The ryT3 target is highlighted in bold type. The underlined space shows the site of a single nucleotide deletion. The substitutions compared to the genomic target sequence are shown in red, and the restriction enzyme recognition sites created by two of them (identified for Oligo F) are in bold type. One additional polymorphism is shown in red and underlined. Oligo R is the complement of Oligo F.

Table S1 Oligonucleotides used in this paper

Oligo Name	Oligo Sequence	Used in
ry-7100-F	CGGCTGTCAGTTTGATGGAGATCG	PCR and sequence for Try1, ry deletion
ry-9532-R	GTAGGCTGACATTGAACTCCCCG	PCR for Try2,Try3, ry deletion
ry-9085-f	GGAATGGCCGTACTGGATGCGTG	PCR for Try2, Try3
ry-9121-fwd	GGCACCTGGAAGGAGTGGATCAACAA	sequencing Try2, Try3
ry-1420-R	AGCTATGGCGCCCACTATCTG	PCR for Try1
y-852-F	CGACATATTATGGCCACCAGTCGTTAC	PCR and sequencing for Ty1
y-1353-R	GCCATATAAAACTGTCAACCACTCAATCAG	PCR for Ty1
PSF2-142-F	CTTTCCGAGCCGGTTTTCCCGT	PCR for PSF2A, B
PSF2-154-F	TTTTCCCGTCTTCGTGCCCCT	sequencing PSF2A, B
PSF2-922-R	GCAGTCCTGTTAGATAAGCGCATATCG	PCR for PSF2A, B
PSF2-100-F	GTGAGACCCATTCTGCCCTA	HRMA for PSF2A, B
PSF2-186-R	TAGCATGGAGGTGTCCCTTT	HRMA for PSF2A, B
PSF2C-12-F	ATTTGTTTTGTTTGTTTATAGCAATTATGGATCCTT	HRMA for PSF2C
PSF2C-108-R	CGTTGCTGAAGTTCGGTATTA	HRMA for PSF2C
PSF2D--178-F	GGATGGCCACGCATCT	HRMA for PSF2D
PSF2D-269-R	CTCCTCCTTGATTTCCTCCA	HRMA for PSF2D
PSF2-trunc	GACCC ATTCTGCCCT ATTCCCTGGA CCACATAGCA CGGTACCAGC GCACGGCCAC TTAGGCCTCTCAA AGGGACACCT CCATGCTAAG TGCATCCATG GCAGGCTC	introduce stop codons into PSF2A
SLD5-248-F	TGGAGCTGATGGTCTCCCAG	HRMA for SLD 5 site 1
SLD5-369-R	ACTGGCCATTATGTAGCGCA	HRMA for SLD 5 site 1
SLD5-514-F	TTCCACAAAGTAGCCACCCA	HRMA for SLD 5 site 2
SLD5-600-R	GGCTCATCAGATTGGGCGTCA	HRMA for SLD 5 site 2
SLD5-33-F	GTTAGAAATCGATGTCAGCGATGGC	PCR for sequencing SLD5
PCD forward	CAGCAGCTAAGAACAAACGAG	PCR and sequencing PCD
PCD reverse	GCAAATCGATGCTAAGCTGAG	PCR PCD
CG12200 forward	GGCGCATATGTTTCGAGTGCATTGC	PCR and sequencing CG12200
CG12200 reverse	GCGGCTCGAGCTAAAGACGAAAGCCTTTAG	PCR CG12200

Table S2 TALEN target sequences.

TALENs	Target	L	S	R
ryT1	tCCGGACCAGTATATCGTTGCT TTTAAGCAGGCCAGA AGAAGGGATGATGACa	21	15	15
ryT2	tCCCAATGCTCGCACCT ATAGCTACTACACGA ATGGCGTGGGAGTCACTGTGGTAGAGa	16	15	26
ryT3	tCGCACCTATAGCTACT ACACGAATGGCGTGGG AGTCACTGTGGTAGAGa	16	16	16
yT1	tCCCGAATACCCGACT AAAGGACCAAGCTCTGGCT AGTGGAGATTATATTCCGCa	15	19	19
yT2	tGCGCCAACAGTATTACCACTGCCT ACCGCATTAAAGTGGATG AGTGTGGTCGGCTGTGGGTTTTGGa	24	18	24
Psf2 A	tCCCTGGACCACATAGCACGGT ACCAGCGCACGGCC ACTGCGTCTCAAAGGGa	21	14	16
Psf2 B	tATTCCCTGGACCACAT AGCACGGTACCAGCGCACGGCC ACTGCGTCTCAAAGGGa	16	22	16
Psf2 C	tGGATCCTTCAATTAT TGAATTTATTGGCGAAA AATGCATGATCAGCATa	15	17	16
Psf2 D	tCTGCGCAAGCAACAAAAGT GCCGAATTGTACCTCC AGAATGGATGGACATGGa	19	16	17
Sld5 A	tCTGGACAAGAACGATT TCCGAGCGGTGGTGC ACTCCATGGAACTGGAGAGGGTGCGCTa	16	15	27
Sld5 B	tAGCCACCCAGTACAT GCCCAACCAGCAGAG AGGAGAGGCGGAGCAGa	15	15	16
PCD A	tGGTTTATTCCCCACAACT GCAACAACAGCAACGAGT AGAAGGTATTCAAAAGGTGGa	18	18	20
PCD B	tCGCAAATCTCAGTTCT GTATTCGCCAGCAGCG AGAGAAGCAGCACGGGCa	16	16	17
CG12200 A	tCTACCGTCAGTTGCAGCAGCT GGAGCAGAACAAGCGCC AGCTGGAGGGCTTTTGCAAGa	21	17	20
CG12200 B	tCCTCGCTAGAAAAGAACT GCCCGCTATGCGGCC AGGTGCTCAAAGCGATCCAGa	18	15	20
CG7224 A	tCGAGATCAAGGAACCAAA GACGCGCACCGAGAAGCT AATGGCCTTCCAGAAGAa	18	18	17
CG11594 A	tGCCTTCAAGCACTCGCTGCT GAAATATGTGGGTGGTC AGGTTTCGCTGGAAATGGa	20	17	18

TALEN names correspond to those in Table 5 of the main text. In each case the sequences to which individual TALENs were designed are highlighted in yellow, while the spacers between binding sites are not highlighted. The number of base pairs in the left binding site (L), spacer (S) and right binding site (R) are tabulated. In addition, each binding site has a T in the 5' position, which is bound by the -1 TALE module, and these are shown in lower case for each TALEN.

Table S3 Details of TALEN mutagenesis.

Gene	TALENs	HRMA+	Crossed	Yielders	Notes
Psf2	A	14/67	2	1	1
	B	9/52	2	0	2
	C	8/13	2	1	1,3
	D	9/32	2	1	1
Sld5	A	9/64	9	2	
	B	13/16	6	3	1
Pcd	A	ND	31	4	
	B	ND	37	4	
CG12200	A	ND	35	1	
	B	ND	62	5	
CG7224	A	17/24	9	7	
CG11594	A	2/16	2	1	

TALEN mutagenesis at sites assayed molecularly. When G0 flies were tested by HRMA, the number that showed evidence of mutations is given, over the total tested. ND, not done. Crossed indicates the number of G0 flies that were fertile. When HRMA was done, only those testing positive were crossed. Yielders indicates the number of G0 flies that gave at least one mutant. Results from individual injection experiments are reported.

NOTES: 1) Most flies that screened positive were also sterile. Frequently, the animals that did give mutant progeny scored as only weakly mosaic. 2) In cases where injections of TALEN mRNAs at 200 µg/ml were unsuccessful, we repeated the injections at 400 µg/ml; only the latter result is reported. 3) In cases where injections at 200 µg/ml were lethal, injections were repeated at 100 µg/ml; only the latter result is reported. When the dead animals were tested by HRMA, they showed a high level of mosaicism. This may indicate that biallelic disruption of the target gene is lethal.

Table S4   ZFN parameters and activities.

Gene	ZFNs	L (bp)	Spacer (bp)	R (bp)	#GNN	Activity
ry	ryAB	9	6	9	6	+
y	yAB	9	6	9	6	+
bw	bwAB	9	6	9	6	+
coilin	coilAB	9	6	9	5	+
pask	pask1	9	6	9	5	+
	pask2	9	6	9	6	-
Sld5	Sld5AB	9	6	9	4	-
	Sld5CD	9	6	9	5	-
Upf3A	Upf3AB	9	6	9	6	-
CG14898	14898AB	9	5	9	4	-
	14898CD	9	6	9	4	-
CG7224	7224CD	9	6	9	5	-
CG8959	8959AB	9	6	9	6	-
	8959CD	9	6	9	5	-

As indicated, each ZFN monomer had 3 fingers, corresponding to 9 bp on the left (L) and right (R) halves of the target. All the spacers were 6 bp, except for the CG14898CD pair. The number of GNN triplets in each target is given. Activity reflects whether (+) or not (-) mutants were obtained following injection of the indicated pair. The Sld5 AB and CD targets overlap.

Table S5 ZFN target sequences.

ryAB	5'-AGC TAC TAC acgaat GGC GTG GGA-3'<br>3'-TCG ATG ATG tgctta CCG CAC CCT-5'
yAB	GCC TAC CGC attaaa GTG GAT GAG<br>CGG ATG GCG taattt CAC CTA CTC
bwAB	CCC ATC ATC aggcgg GAG GTG GGC<br>GGG TAG TAG tccgcc CTC CAC CCG
coilAB	CAC TCC AGC atgaag GTG GAT CTA<br>GTG AGG TCG tacttc CAC CTA GAT
pask1	AGC CAC ATC tcctcc CTG GCG GAG<br>TCG GTG TAG aggagg GAC CGC CTC
pask2	ATC GGC GAC cttcat GGA GGA GGG<br>TAG CCG CTG gaagta CCT CCT CCC
Sld5AB	CAG CAC ATC ctcaac CAG GAG GAG<br>GTC GTG TAG gagttg GTC CTC CTC
Sld5CD	CAC ATC CTC aaccag GAG GAG AGC<br>GTG TAG GAG ttggtc CTC CTC TCG
Upf3AB	TTC GTC GAC cacaag GGC GTC GAG<br>AAG CAG CTG gtgttc CCG CAG CTC
CG14898AB	CTG ACC CAC cctca GAG GGT CCG<br>GAC TGG GTG ggagt CTC CCA GGC
CG14898CD	TGC GAC TGC acagag GTA AGA TGA<br>ACG CTG ACG tgtctc CAG TCT ACT
CG7224CD	AGC GGT GGC gacatg GTG GTC GAG<br>TCG CCA CCG ctgtac CAC CAG CTC
CG8959AB	ATC TAC TAC tatctg GAA GAT GGT<br>TAG ATG ATG atagac CTT CTA CCA
CG8959CD	TCC CTC ATC gatctg AAC GTG GAT<br>AGG GAG TAG ctagac TTG CAC CTA

All sequences are written with same polarities noted explicitly for ryAB. The triplets to which the zinc fingers were designed are shown in red type.
