# Supplementary material for: Comparing Zinc Finger Nucleases and Transcription Activator-Like Effector Nucleases for Gene Targeting in Drosophila
Source: G3 (Bethesda). 2013 Oct 1;3(10):1717–25. doi: 10.1534/g3.113.007260 (PMC3789796; doi:10.1534/g3.113.007260)
Supplement: Supporting Information [file supp_g3.113.007260_FigureS1.pdf]

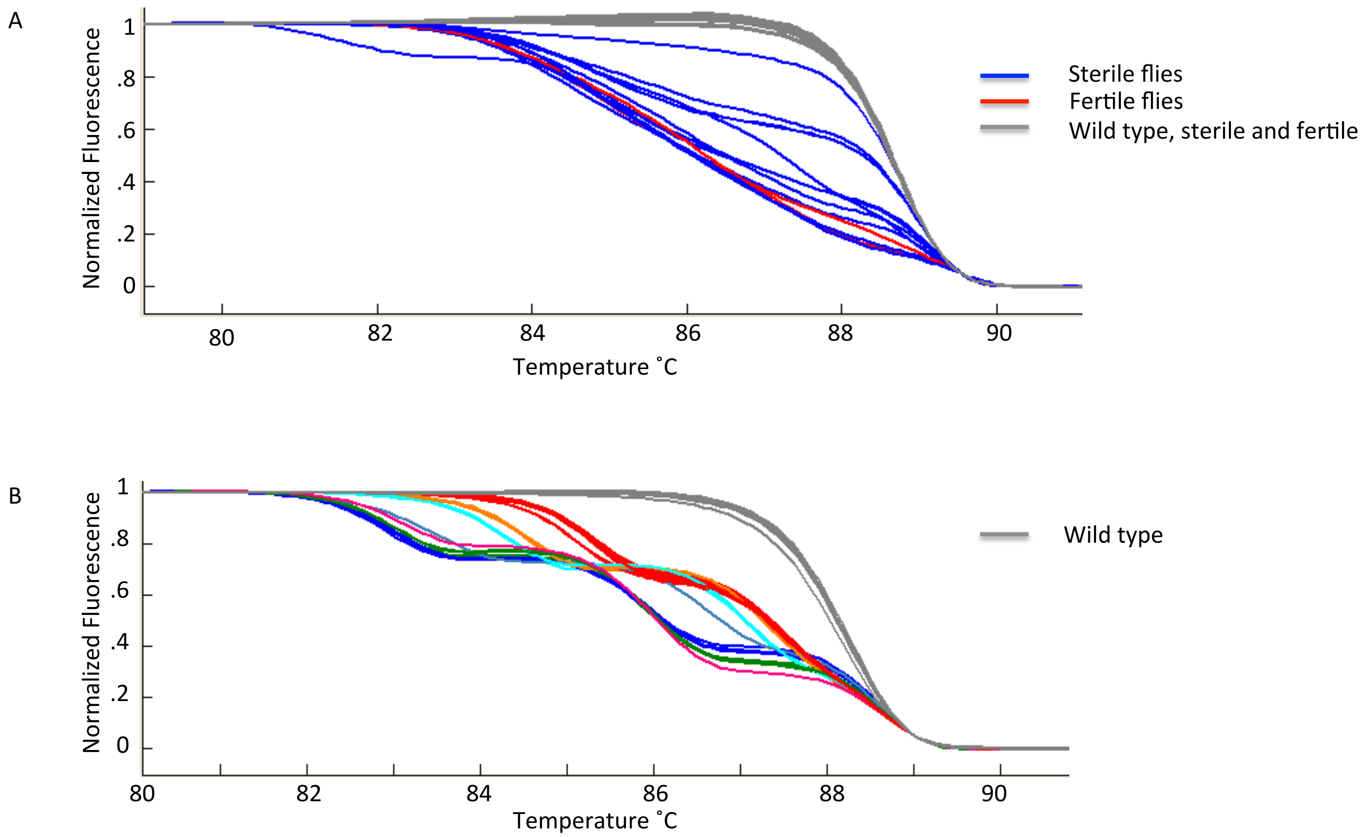

Figure S1 Detecting mutants in *Psf2* with HRMA after mutagenesis with the TALEN pair Psf2A. A. G0 flies were crossed to a balancer stock, allowed to lay for 5-7 days, then collected and tested, along with 8 known wild types. All flies that do not group with the known wild types are considered mutants. In this case, many were sterile, so the 2 fertile vials were kept, and the remainder discarded. All males and virgins from these vials were collected and crossed. B. After being allowed to lay for 5-7 days, the F1 flies were collected and tested in the same way. Each different color represents a unique mutant heterozygote.
