# Supplementary material for: Comparing Zinc Finger Nucleases and Transcription Activator-Like Effector Nucleases for Gene Targeting in Drosophila
Source: G3 (Bethesda). 2013 Oct 1;3(10):1717–25. doi: 10.1534/g3.113.007260 (PMC3789796; doi:10.1534/g3.113.007260)
Supplement: Supporting Information [file supp_g3.113.007260_FigureS2.pdf]

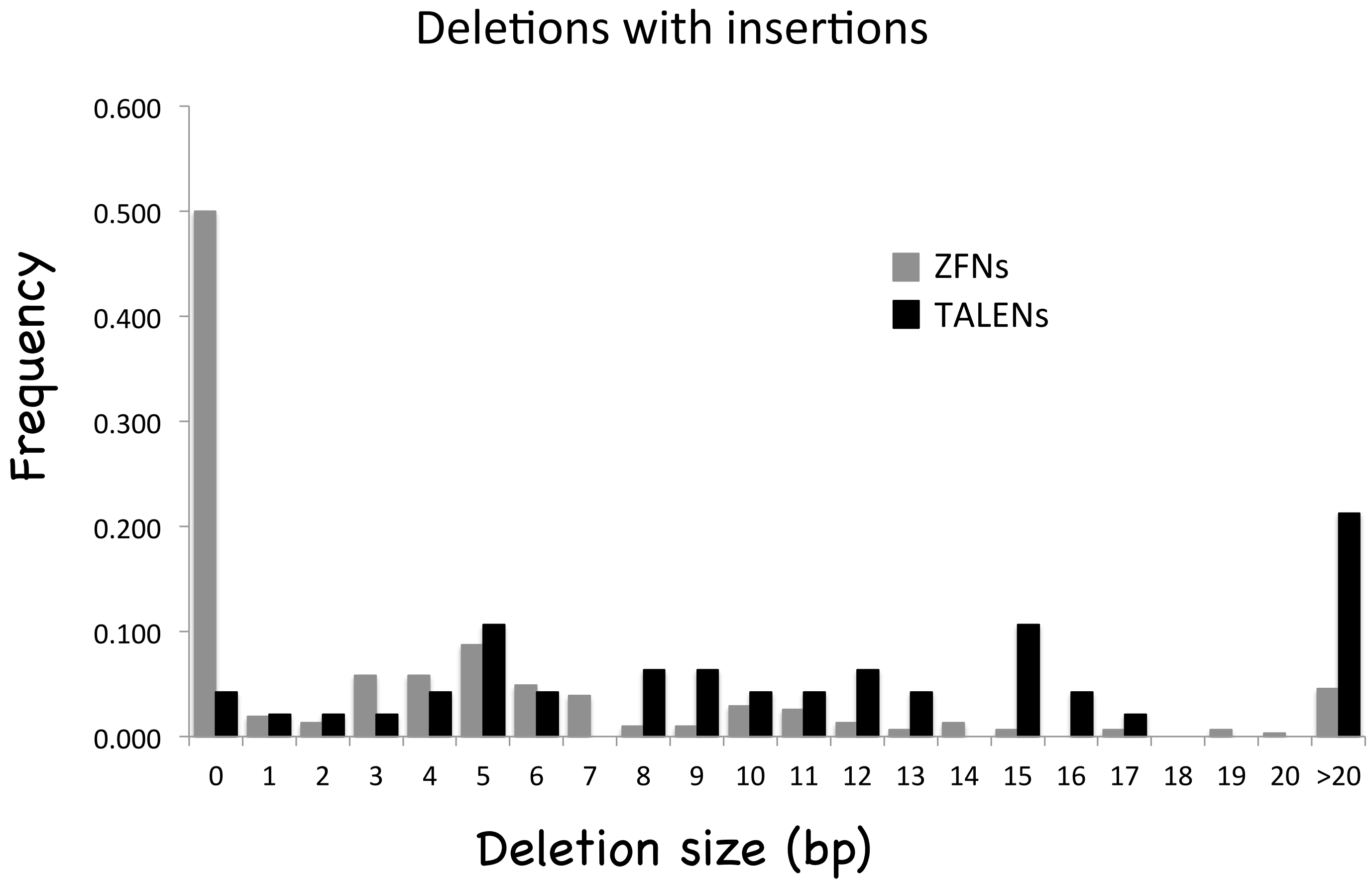

Figure S2 Distribution of the sizes of deletions that are associated with insertions, for ZFNs and TALENs. Data as in Figure 3 of the main text.
