# Supplementary material for: Comparing Zinc Finger Nucleases and Transcription Activator-Like Effector Nucleases for Gene Targeting in Drosophila
Source: G3 (Bethesda). 2013 Oct 1;3(10):1717–25. doi: 10.1534/g3.113.007260 (PMC3789796; doi:10.1534/g3.113.007260)
Supplement: Supporting Information [file supp_g3.113.007260_FigureS4.pdf]

Oligo F *Eco*RI

5'-TTGGATATCACCCGGAAACGAATTCCAATGCC**CGCACCTATAGCTACT**ACAC_AATGGCGT
GGG**AGTCACTGTGGTAGCG**ATCGATTGCCTGACTGGCGACCATCAGGTGCT-3'

*Pvu*I

Oligo R

5'-AGCACCTGATGGTCGCCAGTCAGGCAATCGAT**CGCTACCACAGTGACT**CCCACGCCATT_G
TGT**AGTAGCTATAGGTGCG**GGCATTGGAATTCGTTTCCGGGTGATATCCAA-3'

Figure S4 Sequences of oligonucleotides used as donors with ryT3 TALENs. Each sequence starts at the 5' end. The ryT3 target is highlighted in bold type. The underlined space shows the site of a single nucleotide deletion. The substitutions compared to the genomic target sequence are shown in red, and the restriction enzyme recognition sites created by two of them (identified for Oligo F) are in bold type. One additional polymorphism is shown in red and underlined. Oligo R is the complement of Oligo F.
