# Supplementary material for: Comparing Zinc Finger Nucleases and Transcription Activator-Like Effector Nucleases for Gene Targeting in Drosophila
Source: G3 (Bethesda). 2013 Oct 1;3(10):1717–25. doi: 10.1534/g3.113.007260 (PMC3789796; doi:10.1534/g3.113.007260)
Supplement: Supporting Information [file supp_g3.113.007260_TableS1.pdf]

Table S1 Oligonucleotides used in this paper

Oligo Name	Oligo Sequence	Used in
ry-7100-F	CGGCTGTCAGTTTGATGGAGATCG	PCR and sequence for Try1, ry deletion
ry-9532-R	GTAGGCTGACATTGAACTCCCCG	PCR for Try2,Try3, ry deletion
ry-9085-f	GGAATGGCCGTACTGGATGCGTG	PCR for Try2, Try3
ry-9121-fwd	GGCACCTGGAAGGAGTGGATCAACAA	sequencing Try2, Try3
ry-1420-R	AGCTATGGCGCCCACTATCTG	PCR for Try1
y-852-F	CGACATATTATGGCCACCAGTCGTTAC	PCR and sequencing for Ty1
y-1353-R	GCCATATAAAACTGTCAACCACTCAATCAG	PCR for Ty1
PSF2-142-F	CTTTCCGAGCCGGTTTTCCCGT	PCR for PSF2A, B
PSF2-154-F	TTTTCCCGTCTTCGTGCCCCT	sequencing PSF2A, B
PSF2-922-R	GCAGTCCTGTTAGATAAGCGCATATCG	PCR for PSF2A, B
PSF2-100-F	GTGAGACCCATTCTGCCCTA	HRMA for PSF2A, B
PSF2-186-R	TAGCATGGAGGTGTCCCTTT	HRMA for PSF2A, B
PSF2C-12-F	ATTTGTTTTGTTTGTTTATAGCAATTATGGATCCTT	HRMA for PSF2C
PSF2C-108-R	CGTTGCTGAAGTTCGGTATTA	HRMA for PSF2C
PSF2D--178-F	GGATGGCCACGCATCT	HRMA for PSF2D
PSF2D-269-R	CTCCTCCTTGATTTCCTCCA	HRMA for PSF2D
PSF2-trunc	GACCC ATTCTGCCCT ATTCCCTGGA CCACATAGCA CGGTACCAGC GCACGGCCAC TTAGGCCTCTCAA AGGGACACCT CCATGCTAAG TGCATCCATG GCAGGCTC	introduce stop codons into PSF2A
SLD5-248-F	TGGAGCTGATGGTCTCCCAG	HRMA for SLD 5 site 1
SLD5-369-R	ACTGGCCATTATGTAGCGCA	HRMA for SLD 5 site 1
SLD5-514-F	TTCCACAAAGTAGCCACCCA	HRMA for SLD 5 site 2
SLD5-600-R	GGCTCATCAGATTGGGCGTCA	HRMA for SLD 5 site 2
SLD5-33-F	GTTAGAAATCGATGTCAGCGATGGC	PCR for sequencing SLD5
PCD forward	CAGCAGCTAAGAACAAACGAG	PCR and sequencing PCD
PCD reverse	GCAAATCGATGCTAAGCTGAG	PCR PCD
CG12200 forward	GGCGCATATGTTTCGAGTGCATTGC	PCR and sequencing CG12200
CG12200 reverse	GCGGCTCGAGCTAAAGACGAAAGCCTTTAG	PCR CG12200
