# Supplementary material for: Comparing Zinc Finger Nucleases and Transcription Activator-Like Effector Nucleases for Gene Targeting in Drosophila
Source: G3 (Bethesda). 2013 Oct 1;3(10):1717–25. doi: 10.1534/g3.113.007260 (PMC3789796; doi:10.1534/g3.113.007260)
Supplement: Supporting Information [file supp_g3.113.007260_TableS2.pdf]

Table S2 TALEN target sequences.

TALENs	Target	L	S	R
ryT1	tCCGGACCAGTATATCGTTGCT TTTAAGCAGGCCAGA AGAAGGGATGATGACa	21	15	15
ryT2	tCCCAATGCTCGCACCT ATAGCTACTACACGA ATGGCGTGGGAGTCACTGTGGTAGAGa	16	15	26
ryT3	tCGCACCTATAGCTACT ACACGAATGGCGTGGG AGTCACTGTGGTAGAGa	16	16	16
yT1	tCCCGAATACCCGACT AAAGGACCAAGCTCTGGCT AGTGGAGATTATATTCCGCa	15	19	19
yT2	tGCGCCAACAGTATTACCACTGCCT ACCGCATTAAAGTGGATG AGTGTGGTCGGCTGTGGGTTTTGGa	24	18	24
Psf2 A	tCCCTGGACCACATAGCACGGT ACCAGCGCACGGCC ACTGCGTCTCAAAGGGa	21	14	16
Psf2 B	tATTCCCTGGACCACAT AGCACGGTACCAGCGCACGGCC ACTGCGTCTCAAAGGGa	16	22	16
Psf2 C	tGGATCCTTCAATTAT TGAATTTATTGGCGAAA AATGCATGATCAGCATa	15	17	16
Psf2 D	tCTGCGCAAGCAACAAAAGT GCCGAATTGTACCTCC AGAATGGATGGACATGGa	19	16	17
Sld5 A	tCTGGACAAGAACGATT TCCGAGCGGTGGTGC ACTCCATGGAACTGGAGAGGGTGCGCTa	16	15	27
Sld5 B	tAGCCACCCAGTACAT GCCCAACCAGCAGAG AGGAGAGGCGGAGCAGa	15	15	16
PCD A	tGGTTTATTCCCCACAACT GCAACAACAGCAACGAGT AGAAGGTATTCAAAAGGTGGa	18	18	20
PCD B	tCGCAAATCTCAGTTCT GTATTCGCCAGCAGCG AGAGAAGCAGCACGGGCa	16	16	17
CG12200 A	tCTACCGTCAGTTGCAGCAGCT GGAGCAGAACAAGCGCC AGCTGGAGGGCTTTTGCAAGa	21	17	20
CG12200 B	tCCTCGCTAGAAAAGAACT GCCCGCTATGCGGCC AGGTGCTCAAAGCGATCCAGa	18	15	20
CG7224 A	tCGAGATCAAGGAACCAAA GACGCGCACCGAGAAGCT AATGGCCTTCCAGAAGAa	18	18	17
CG11594 A	tGCCTTCAAGCACTCGCTGCT GAAATATGTGGGTGGTC AGGTTTCGCTGGAAATGGa	20	17	18

TALEN names correspond to those in Table 5 of the main text. In each case the sequences to which individual TALENs were designed are highlighted in yellow, while the spacers between binding sites are not highlighted. The number of base pairs in the left binding site (L), spacer (S) and right binding site (R) are tabulated. In addition, each binding site has a T in the 5’ position, which is bound by the -1 TALE module, and these are shown in lower case for each TALEN.
