# Supplementary material for: Comparing Zinc Finger Nucleases and Transcription Activator-Like Effector Nucleases for Gene Targeting in Drosophila
Source: G3 (Bethesda). 2013 Oct 1;3(10):1717–25. doi: 10.1534/g3.113.007260 (PMC3789796; doi:10.1534/g3.113.007260)
Supplement: Supporting Information [file supp_g3.113.007260_TableS3.pdf]

Table S3 Details of TALEN mutagenesis.

Gene	TALENs	HRMA+	Crossed	Yielders	Notes
Psf2	A	14/67	2	1	1
	B	9/52	2	0	2
	C	8/13	2	1	1,3
	D	9/32	2	1	1
Sld5	A	9/64	9	2	
	B	13/16	6	3	1
Pcd	A	ND	31	4	
	B	ND	37	4	
CG12200	A	ND	35	1	
	B	ND	62	5	
CG7224	A	17/24	9	7	
CG11594	A	2/16	2	1	

TALEN mutagenesis at sites assayed molecularly. When G0 flies were tested by HRMA, the number that showed evidence of mutations is given, over the total tested. ND, not done. Crossed indicates the number of G0 flies that were fertile. When HRMA was done, only those testing positive were crossed. Yielders indicates the number of G0 flies that gave at least one mutant. Results from individual injection experiments are reported.

NOTES: 1) Most flies that screened positive were also sterile. Frequently, the animals that did give mutant progeny scored as only weakly mosaic. 2) In cases where injections of TALEN mRNAs at 200 µg/ml were unsuccessful, we repeated the injections at 400 µg/ml; only the latter result is reported. 3) In cases where injections at 200 µg/ml were lethal, injections were repeated at 100 µg/ml; only the latter result is reported. When the dead animals were tested by HRMA, they showed a high level of mosaicism. This may indicate that biallelic disruption of the target gene is lethal.
