# Supplementary material for: Comparing Zinc Finger Nucleases and Transcription Activator-Like Effector Nucleases for Gene Targeting in Drosophila
Source: G3 (Bethesda). 2013 Oct 1;3(10):1717–25. doi: 10.1534/g3.113.007260 (PMC3789796; doi:10.1534/g3.113.007260)
Supplement: Supporting Information [file supp_g3.113.007260_TableS4.pdf]

Table S4   ZFN parameters and activities.

Gene	ZFNs	L (bp)	Spacer (bp)	R (bp)	#GNN	Activity
ry	ryAB	9	6	9	6	+
y	yAB	9	6	9	6	+
bw	bwAB	9	6	9	6	+
coilin	coilAB	9	6	9	5	+
pask	pask1	9	6	9	5	+
	pask2	9	6	9	6	-
Sld5	Sld5AB	9	6	9	4	-
	Sld5CD	9	6	9	5	-
Upf3A	Upf3AB	9	6	9	6	-
CG14898	14898AB	9	5	9	4	-
	14898CD	9	6	9	4	-
CG7224	7224CD	9	6	9	5	-
CG8959	8959AB	9	6	9	6	-
	8959CD	9	6	9	5	-

As indicated, each ZFN monomer had 3 fingers, corresponding to 9 bp on the left (L) and right (R) halves of the target. All the spacers were 6 bp, except for the CG14898CD pair. The number of GNN triplets in each target is given. Activity reflects whether (+) or not (-) mutants were obtained following injection of the indicated pair. The Sld5 AB and CD targets overlap.
