# Supplementary material for: Comparing Zinc Finger Nucleases and Transcription Activator-Like Effector Nucleases for Gene Targeting in Drosophila
Source: G3 (Bethesda). 2013 Oct 1;3(10):1717–25. doi: 10.1534/g3.113.007260 (PMC3789796; doi:10.1534/g3.113.007260)
Supplement: Supporting Information [file supp_g3.113.007260_TableS5.pdf]

Table S5 ZFN target sequences.

ryAB	5'-AGC TAC TAC acgaat GGC GTG GGA-3'<br>3'-TCG ATG ATG tgctta CCG CAC CCT-5'
yAB	GCC TAC CGC attaaa GTG GAT GAG<br>CGG ATG GCG taattt CAC CTA CTC
bwAB	CCC ATC ATC aggcgg GAG GTG GGC<br>GGG TAG TAG tccgcc CTC CAC CCG
coilAB	CAC TCC AGC atgaag GTG GAT CTA<br>GTG AGG TCG tacttc CAC CTA GAT
pask1	AGC CAC ATC tcctcc CTG GCG GAG<br>TCG GTG TAG aggagg GAC CGC CTC
pask2	ATC GGC GAC cttcat GGA GGA GGG<br>TAG CCG CTG gaagta CCT CCT CCC
Sld5AB	CAG CAC ATC ctcaac CAG GAG GAG<br>GTC GTG TAG gagttg GTC CTC CTC
Sld5CD	CAC ATC CTC aaccag GAG GAG AGC<br>GTG TAG GAG ttggtc CTC CTC TCG
Upf3AB	TTC GTC GAC cacaag GGC GTC GAG<br>AAG CAG CTG gtgttc CCG CAG CTC
CG14898AB	CTG ACC CAC cctca GAG GGT CCG<br>GAC TGG GTG ggagt CTC CCA GGC
CG14898CD	TGC GAC TGC acagag GTA AGA TGA<br>ACG CTG ACG tgtctc CAG TCT ACT
CG7224CD	AGC GGT GGC gacatg GTG GTC GAG<br>TCG CCA CCG ctgtac CAC CAG CTC
CG8959AB	ATC TAC TAC tatctg GAA GAT GGT<br>TAG ATG ATG atagac CTT CTA CCA
CG8959CD	TCC CTC ATC gatctg AAC GTG GAT<br>AGG GAG TAG ctagac TTG CAC CTA

All sequences are written with same polarities noted explicitly for ryAB. The triplets to which the zinc fingers were designed are shown in red type.
